# Carnitine palmitoyltransferase II (CPT II) deficiency responsible for refractory cardiac arrhythmias, acute multiorgan failure and early fatal outcome

**DOI:** 10.1186/s13052-024-01632-x

**Published:** 2024-04-14

**Authors:** Gregorio Serra, Vincenzo Antona, Vincenzo Insinga, Giusy Morgante, Alessia Vassallo, Simona La Placa, Ettore Piro, Sergio Salerno, Ingrid Anne Mandy Schierz, Eloisa Gitto, Mario Giuffrè, Giovanni Corsello

**Affiliations:** 1 Department of Health Promotion, Mother and Child Care, Internal Medicine and Medical Specialties “Giuseppe D’Alessandro”, University of Palermo, Palermo, Italy; 2 Department of Human Pathology in Adult and Developmental Age “Gaetano Barresi”, University of Messina, Messina, Italy

**Keywords:** CPTII, Neonatal screening, Next generation sequencing, Fatty acid oxidation defect

## Abstract

**Background:**

Carnitine palmitoyltransferase II (CPT II) deficiency is a rare inborn error of mitochondrial fatty acid metabolism with autosomal recessive pattern of inheritance. Its phenotype is highly variable (neonatal, infantile, and adult onset) on the base of mutations of the *CPT II* gene. In affected subjects, long-chain acylcarnitines cannot be subdivided into carnitine and acyl-CoA, leading to their toxic accumulation in different organs. Neonatal form is the most severe, and all the reported patients died within a few days to 6 months after birth. Hereby, we report on a male late-preterm newborn who presented refractory cardiac arrhythmias and acute multiorgan (hepatic, renal, muscular) injury, leading to cerebral hemorrhage, hydrocephalus, cardiovascular failure and early (day 5 of life) to death. Subsequently, extended metabolic screening and target next generation sequencing (NGS) analysis allowed the CPT II deficiency diagnosis.

**Case presentation:**

The male proband was born at 36^+ 4^ weeks of gestation by spontaneous vaginal delivery. Parents were healthy and nonconsanguineous, although both coming from Nigeria. Family history was unremarkable. Apgar score was 9/9. At birth, anthropometric measures were as follows: weight 2850 g (47th centile, -0.07 standard deviations, SD), length 50 cm (81st centile, + 0.89 SD) and occipitofrontal circumference (OFC) 35 cm (87th centile, + 1.14 SD). On day 2 of life our newborn showed bradycardia (heart rate around 80 bpm) and hypotonia, and was then transferred to the Neonatal Intensive Care Unit (NICU). There, he subsequently manifested many episodes of ventricular tachycardia, which were treated with pharmacological (magnesium sulfate) and electrical cardioversion. Due to the critical conditions of the baby (hepatic, renal and cardiac dysfunctions) and to guarantee optimal management of the arrythmias, he was transferred to the Pediatric Cardiology Reference Center of our region (Sicily, Italy), where he died 2 days later. Thereafter, the carnitines profile evidenced by the extended metabolic screening resulted compatible with a fatty acid oxidation defect (increased levels of acylcarnitines C_16_ and C_18_, and low of C_2_); afterwards, the targeted next generation sequencing (NGS) analysis revealed the known c.680 C > T p. (Pro227Leu) homozygous missense mutation of the *CPTII* gene, for diagnosis of CPT II deficiency. Genetic investigations have been, then, extended to the baby’s parents, who were identified as heterozygous carriers of the same variant. When we meet again the parents for genetic counseling, the mother was within the first trimester of her second pregnancy. Therefore, we offered to the couple and performed the prenatal target NGS analysis on chorionic villi sample, which did not detect any alterations, excluding thus the CPT II deficiency in their second child.

**Conclusions:**

CPTII deficiency may be suspected in newborns showing cardiac arrhythmias, associated or not with hypertrophic cardiomyopathy, polycystic kidneys, brain malformations, hepatomegaly. Its diagnosis should be even more suspected and investigated in cases of increased plasmatic levels of creatine phosphokinase and acylcarnitines in addition to kidney, heart and liver dysfunctions, as occurred in the present patient. Accurate family history, extended metabolic screening, and multidisciplinary approach are necessary for diagnosis and adequate management of affected subjects. Next generation sequencing (NGS) techniques allow the identification of the *CPTII* gene mutation, essential to confirm the diagnosis before or after birth, as well as to calculate the recurrence risk for family members. Our report broads the knowledge of the genetic and molecular bases of such rare disease, improving its clinical characterization, and provides useful indications for the treatment of patients.

## Background

Carnitine palmitoyltransferase II (CPT II) deficiency is a rare genetic metabolic disorder, and around 350 cases have been reported to date [1]. Three forms of the disease have been described: the lethal neonatal form, the severe infantile hepatocardiomuscular type, and the myopathic adult onset one [2]. The latter primarily involves the skeletal muscle. Conversely, the clinical manifestations of the severe infantile form include hepatomegaly, cardiac abnormalities, hypoketotic hypoglycemia, elevated liver enzymes and seizures [2]. The lethal neonatal type shows symptoms of the infantile disease as well as dysmorphic features/birth defects, including microcephaly, kidneys and brain malformations. Congenital abnormalities of such lethal form, associated with the homozygote c.680 C *>* T (p.Pro227Leu) mutation of the *CPTII* gene, were firstly described in 1994 [3]. Indeed, proline could be crucial for protein conformation, and its substitution in essential domains may be responsible for relevant enzyme or transporter dysfunction, and then for severe clinical manifestations of disease [4]. CPT II deficiency can be suspected by acylcarnitine analysis in blood, revealing a typical profile with accumulation of long-chain species [5]. Hereby, we describe the particularly complex neonatological management of a late-preterm newborn presenting with upcoming episodes of cardiac arrythmias, requiring advanced resuscitation maneuvers and refractory to pharmacological and electrical cardioversion, in addition to multiorgan dysfunction, which led the patient to be transferred to the Pediatric Cardiology Reference Center of our region (Sicily, Italy), where he died due to cardiocirculatory failure two days later. The results of the extended metabolic screening showed a metabolic profile compatible with fatty-acid oxidation defect, which was subsequently confirmed and defined as CPTII deficiency by target next generation sequencing analysis.

## Case presentation

A male newborn was delivered at 36^+ 4^ weeks of the first pregnancy, by spontaneous vaginal delivery. Current gestation has not been followed by either gynecologists or other health professionals, and thus obstetric information were not available. Parents were healthy and nonconsanguineous, although both coming from Nigeria. Family history was unremarkable. Apgar score was 9/9. At birth, anthropometric measures were as follows: weight 2850 g (47th centile, -0.07 standard deviations, SD), length 50 cm (81st centile, + 0.89 SD) and occipitofrontal circumference (OFC) 35 cm (87th centile, + 1.14 SD). Clinical examination did not show either congenital anomalies or any dysmorphic features. On day 2 of life bradycardia (heart rate [HR] 80 beats per minute, bpm) and central-type hypotonia were noted. Then, he was transferred to the Neonatal Intensive Care Unit (NICU), where acute kidney failure (plasmatic creatinine 1.23 mg/dL, normal values [n.v.] 0.24–0.85; blood urea nitrogen 81 mg/dL, n.v. 3–25), and hepatic injury (AST 138 IU/L, n.v. 15–60; ALT 11 IU/L, n.v. 5–25), along with hyperkalemia (7.4 mEq/L, n.v. 3.5-5), and increase of serum cardiac enzymes (myoglobin 113 mcg/L, n.v. 6–85; troponin T 673 ng/L, n.v. <100) and of the other cytolysis indexes (creatine phosphokinase, CPK, 559 IU/L, n.v. <150; lactic dehydrogenase, LDH, 773 IU/L, n.v. 160–450) were observed (Table 1).

**Table 1:** Hematochemical profile of our patient

	Admission to NICU (day 2)	Transfer to the Pediatric Cardiology Center (day 3)
K mEq/L	8.1	7.4
Creatinine mg/dL	1.23	1.42
Urea nitrogen mg/dL	81	99
AST/ALT IU/L	64/9	88/14
CPK IU/L		558
Myoglobin mcg/L		113
Troponin mcg/L		673

**Fig. 1:**
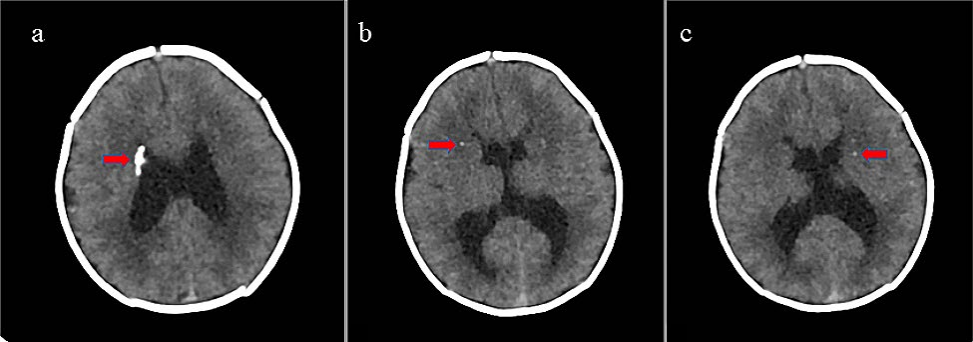
Brain computed tomography of present patient: hydrocephalus associated with subependymal calcification (**a**), and multiple punctate intraparenchymal hemorrhages (**b** and **c**) indicated by arrows

Abdomen ultrasound (US) examination showed bilateral nephromegaly (kidneys length evaluated according with pediatric radiology reference values for preterm babies [6]) and renal diffuse parenchymal increase of echogenicity, as well as right hydronephrosis. Intravenous (i.v.) rehydration with glucose and sodium chloride was then started, also due to lethargy and difficulties of enteral feeding, along with sodium bicarbonate and ion exchange resins administration for the correction of metabolic acidosis and hyperkalemia, respectively. In the meanwhile, a continuous electrocardiographic monitoring was also begun, disclosing a self-resolving episode of ventricular tachycardia. Heart US did not reveal any cardiac structural anomalies, except for a small patent *foramen ovale*. Thereafter, a crisis characterized by pallor, reduction of peripheral oxygen saturation levels, and asystole occurred, from which he recovered after advanced resuscitation maneuvers including intubation, positive pressure ventilation (PPV), chest compressions and i.v. adrenalin administration. Subsequently, while being supported through invasive mechanical ventilation, he presented several episodes of pulseless ventricular tachycardia (HR 250–260 bpm), treated with i.v. bolus infusion of magnesium sulphate (8 mg/kg) and defibrillation (9 Joules per kilogram). Brain US identified periventricular hyperechogenicity and triventricular hydrocephalus. Computed tomography (CT) scan, performed soon after, confirmed triventricular hydrocephalus, and additionally showed subependymal calcification and multiple punctate intraparenchymal hemorrhages (Fig. 1a/b/c). Due to the persistently severe clinical conditions and to guarantee the optimal managing of arrhythmic crises, on day 3 of life the baby was transferred to the Pediatric Cardiology Reference Centre of our region (Sicily, Italy), where he died due to cardiocirculatory failure two days later. Before to be transferred, blood samples were obtained both for expanded metabolic screening (EMS) and genetic testing. The EMS profile showed an increase of the acylcarnitines C_16_ (27.4 µmol/L, n.v. 0.95–8.6) and C_18_ (6.4 µmol/L, n.v. 0.29–2.1), and low levels of C_2_ (2.6 µmol/L, n.v. 3–49) (Table 2), rising the suspicion of a fatty-acid oxidation defect.

**Table 2:** Expanded metabolic screening profile of our newborn

Acylcarnitines	(µmol/L)	Normal values (µmol/L)
C2	2.6	3.0-4.9
C14	1.56	< 0.49
C16	27.4	0.95–8.6
C16:	2.12	<0.51
C16OH	0.4	<0.07
C17	0.4	< 0.09
C18	6.4	0.29–2.1
C18:1	7.41	3.27
OH-C18:1	0.29	< 0.12
C20	0.33	< 0.16

Finally, a few weeks later, the target NGS analysis performed on the *CPT II* and *SLC25A20* genes revealed the c.680 C > T p.(Pro227Leu) (NM_000098) missense mutation of *CPTII*, for CPTII deficiency diagnosis. Such variant has already been reported in literature, in the homozygous state, as associated with the neonatal form of CPTII deficiency [2]. Conversely, no variants were found on the *SLC25A20* gene, whose mutations are associated with the carnitine-acylcarnitine translocase (CACT) deficiency (MIM 212,138), which is as well a rare autosomal recessive disorder of long-chain fatty acid oxidation [7]. The bioinformatic prediction tools used indicate this mutation as potentially pathogenic (Mutation Taster; SIFT; Polyphen-2), according with the guidelines of the American College of Medical Genetics (ACMG) Laboratory Practice Committee Working Group [8]. The analysis has been performed through targeted-resequencing of exons and splicing sites of the genes under investigation, with protocol Paired-End 150 bp carried out on MiSeq (Illumina, USA). Sequencing has been preceded by selective enrichment of the DNA regions of interest through their hybridization with a specifically designed (Nextera, Illumina) set of probes. The reads obtained by sequencing analysis have been aligned with the reference genome (hg19/b37). The qualitative parameters applied were the following: >95% of targeted bases with coverage > 15X, and 85% >40X, mean coverage > 100X. The data obtained by NGS have been analyzed through CoNVaDING (Copy Number Variation Detection in Next-generation sequencing Gene panels) tool [9]. The genetic test was then performed on both parents, and identified the same heterozygous variant found in their son. When we meet again the parents for genetic counseling, the mother was within the first trimester of her second pregnancy. Therefore, we offered to the couple the prenatal target NGS analysis on chorionic villi sample. Such invasive genetic investigation did not disclose any alterations, excluding thus CPT II deficiency in their second child.

## Discussion and conclusions

Carnitine palmitoyltransferase II (CPTII) deficiency is a rare autosomal recessive disorder of mitochondrial fatty acid oxidation, due to biallelic pathogenetic variants in *CPT* gene, whose locus is in 1p32.3 [1]. Based on the type of variants, the disease may be classified into three different phenotypes: the adult myopathic form (MIM 255,110), with rhabdomyolysis following stress, infection, prolonged exercise, fasting, and appearing in adulthood; a sever infantile type (MIM 600,649), with hepatocardiomuscular involvement and including hypoketotic hypoglycemia, increased transaminases, cardiomyopathy, hepatomegaly, vomiting and seizures; and the lethal neonatal one (MIM 608,836), with liver failure, hypoketotic hypoglycemia, cardiomyopathy with arrhythmias, seizures and coma after fasting or infection, in addition to dysmorphic features and/or organ malformations such as cystic renal dysplasia and brain dysgenesis [2, 10, 11–13]. Today, around 20 cases affected with the lethal neonatal form, 30 with the severe infantile hepatocardiomuscular one, and 300 with the myopathic phenotype have been reported [14]. However, the prevalence of CPTII deficiency may be underestimated, as it is often responsible for abortion, and for the frequent association of the myopathic form with mild symptoms, not always allowing clinicians to formulate the diagnosis [14].

The multisystem involvement of the affected subjects is due to the role CPTII has in the lipidic metabolism of many organs like brain, kidneys, and heart. The carnitine palmitoyltransferase enzyme system (CPT), in association with acyl-CoA synthetase and carnitine-acylcarnitine translocase, allows long-chain fatty acids (LCFA) to be converted into AcylCoA esters, and then to reach the mitochondrial matrix for β-oxidation [5]. However, the mitochondrial membrane is not permeable to the AcylCoA esters, and only the carnitine shuttle with the CPI (carnitine palmitoyltransferase I), CACT (carnitine-acylcarnitine translocase) and CPTII proteins, allows them to pass in the mitochondrion. In the inner mitochondrial membrane, CPTII promotes the transformation of acylcarnitine in free carnitine and Acyl-CoA, which is used for fatty acids beta-oxidation. Thus, a CPTII deficiency is associated with a build-up of toxic long-chain acyl-CoA or acylcarnitine esters in organs and tissues, with consequent functional and morphological changes in tissues, and a reduced capacity for fatty acid beta-oxidation [15–18].

The neonatal form of CPT II deficiency is lethal. In most of cases, newborns die in the first few hours of life, and only two patients who lived besides 30 days have been reported in literature [2, 3, 14, 15]. During pregnancy, US findings rising the suspicion of CPTII deficiency include oligohydramnios and fetal malformations [19]. In present patient we have no data about pregnancy, as the proband’s mother did not perform any prenatal care and investigations.

The typical metabolic disturbance of disease is non-ketotic hypoglycemia (however absent in our newborn) due to decreased fatty acid beta-oxidation, along with metabolic acidosis and hyperkalemia (both conversely documented). Cardiomegaly is observed in 76% of cases, while cardiac arrhythmias, which were the main clinical manifestations of our patient, is present in 62% of affected subjects. Polycystic kidneys are found in about half (54%) of cases [14, 15, 20]. Present patient did not show any dysmorphic features (microcephaly, long-tapered fingers or hypoplastic toenails), while the abnormalities documented on the brain CT scan (triventricular hydrocephalus associated with subependymal calcification and multiple punctate intraparenchymal hemorrhages, Fig. 1a/b/c) might be related with the occurrence of injuries in various stages of brain development. A relevant role may also be played by the repetitive arrhythmic crises requiring resuscitation and cardioversion. CT is the imaging modality of choose in emergency for unstable patients, and therefore it was deemed to be performed in the present case [20, 21]. In present newborn, despite the cardiac dysfunction, heart US did not evidence cardiomegaly or cardiomyopathy, while abdominal US disclosed bilateral nephromegaly and renal diffuse parenchymal increase of echogenicity, according with literature reports [11].

CPTII deficiency can also lead to increased levels of serum transaminases, CPK, creatinine, urea nitrogen, myoglobin, and ammonia [5, 14, 19], as well as to organic aciduria and increased urinary elimination of long-chain bicarboxylic acids [5]. Moreover, in neonatal forms, plasmatic long-chain acylcarnitines (C_16_ and C_18:1_) levels are increased, with low values of C_2_ and higher free acylcarnitine/carnitine ratio [5, 10, 14]. Actually, the most sensitive indicator for diagnosing CPT-II deficiency is a high (C_16_ + C_18:1_)/C_2_ ratio [22, 23]. In the present patient, in accordance with previous studies, haematochemical examinations showed increased renal and hepatic function tests along with cytolysis indexes (Table 1) [11]. Furthermore, metabolic screening typically showed high levels of the acylcarnitines C_16_ (27.4 µmol/L, n.v. 0.95–8.6) and C_18_ (6.4 µmol/L, n.v. 0.29–2.1), and decreased of C_2_ (2.6 µmol/L, n.v. 3–49) (Table 2), suggesting a fatty acid oxidation defect.

A definite CPT II deficiency diagnosis may be obtained only by genetic investigations [14]. More than 90 mutations have been reported to date, most of them being missense variants or small deletions [11, 14]. A single-gene testing or a multigene panel may be applied. In present report, the pathogenic 680 C > T missense variant of the *CPTII* gene was found in homozygosity (Table 3**)**. This mutation is associated with the fatal neonatal form of CPTII deficiency [2]. Clinical features and outcomes, along with consistent genetic variants of previously reported patients affected with the neonatal lethal form, are summarized in Table 3.

**Table 3:** Comparison between our proband and previously reported patients with neonatal lethal form of CPTII deficiency

Authors	Genetic variants	Clinical features	Laboratory tests	Enzymatic activity	Day of exitus
Taroni et al., 1994 [3]	c.680 C> T p.(Pro227Leu)	Respiratory distress Cardiac arrhythmia Heart failure Cardiomyopathy Polymicrogyria Intraventricular haemorrhage		<15%	4
Smeets et al., 2003 [24]	c.534_558del25insT and c.520G> A p.(Leu178_Ile186delinsPhe) and p.(Glu174Lys) (compound heterozygosity)	Lethargy Cardiac arrhythmias Cardiomegaly		2%	10
Isackson et al., 2008 [2]	c.680 C> T p.(Pro227Leu) c.164 C> G p.(Pro55Arg) c.359 A> G p.(Tyr120Cys) (each mutation for a different patient)	Hypothermia Seizures Polycystic kidney Periventricular calcifications Hypoketotic hypoglycaemia Ventricular arrhythmia	Acylcarnitines		3 to 14
Elpeleg et al., 2001 [25]	c.1237 A> G p.(Phe448Leu)	Corpus callosum agenesis Ventriculomegaly Polycystic kidneys/tubular lipidosis Cardiomegaly Hepatic steatosis (autoptic findings)		Unknown	Abortion
Hissink-Muller et al., 2009 [15]	c.680 C> T p.(Pro227Leu)	Cardiomegaly Cardiac arrhythmias	Acylcarnitines	Unknown	196
Present report	c.680 C> T p.(Pro227Leu)	Renal insufficiency Nephromegaly Kidney hyperechogenicity Cardiac arrhythmias Hydrocephalus Encephalic subependymal calcification and parenchymal haemorrhagic *foci*	Acylcarnitines Metabolic acidosis Hyperkalemia Increased cardiac enzymes Hypertransaminasemia	Unknown	5

The main variants associated with the hepatocardiomuscular and/or myopathic forms are listed in Table 4. The former, although showing autosomal recessive pattern of inheritance, may also be due to compound heterozygous mutations, which seem to be linked with milder phenotypes appearing later in life [11, 14].

**Table 4:** Clinical forms of CPTII deficiency and main pathogenic reported variants of the *CPTII* gene (modified by Wieser T., 2019 [14])

Clinical form	Main pathogenic variants of *CPTII*
Neonatal lethal (AR)	c.680 C > T (p.Pro227Leu)
Hepatocardiomuscular infantile (AR/compound heterozygosity)	c.359 A > G (p.Tyr120Cys) c.1507 C > T (p.Arg503Cys) c.1883 A > C (p.Tyr628Ser) c.1891 C > T (p.Arg631Cys)
Myopathic (AR/AD)	c.338 C > T (p.Ser113Leu) c.149 C > A (p.Pro50His)

AR = autosomal recessive; AD = autosomal dominant

When the CPTII deficiency is diagnosed, glucose solution administration is required. Intravenous glucose infusion can improve exercise tolerance, as it spares muscle glycogenolysis, while stress or fasting should be avoided [5, 14].

CPTII deficiency may be suspected in newborns showing cardiac arrhythmias, associated or not with hypertrophic cardiomyopathy, polycystic kidneys, brain malformations, hepatomegaly. Moreover, in cases with increased plasmatic levels of creatine phosphokinase and acylcarnitines in addition to kidney, heart, and liver dysfunctions, CPTII deficiency must be investigated. Accurate family history, extended metabolic screening, and multidisciplinary approach are necessary for early diagnosis and management of patients. In the present proband, NGS techniques, which included *CPTII* and *SLC25A20* genes analysis, allowed the identification of the c.680 C > T p.(Pro227Leu) homozygous variant of *CPTII*, and of the same heterozygous mutation in his parents. Gene sequencing is essential in affected subjects and healthy carriers, since it may suggest genotype-phenotype correlations and aid clinicians to provide precise reproductive counseling, also in view of primary and/or secondary prevention of the disease [25–47]. Indeed, NGS may be crucial for prenatal diagnosis, which can be performed on amniotic fluid or chorionic villi [14]. Furthermore, it may address pediatricians towards suitable individualized approaches, avoiding disproportionate treatments in cases with lethal neonatal forms, as well as reassuring families in those with milder adult-onset phenotypes and favorable evolution [48–58]. The present report may broaden the knowledge of the genetic and molecular basis of CPTII deficiency, improving its clinical characterization and providing indications for the treatment of patients.

## Data Availability

The datasets used and analyzed during the current study are available from the corresponding author on reasonable request.

## Abbreviations

AC: Acylcarnitines
Bpm: beats per minute
CPK: Creatin Phosphokinase
CPTII: Carnitine palmitoyltransferase II
CT: computed tomography
EMS: expanded metabolic screening
HR: Heart rate
LCFA: Long-chain fatty acids
NGS: Next Generation Sequencing
NICU: Neonatal intensive Care Unit
US: Ultrasound
